# Lycopene supplementation reduces inflammatory, histopathological and DNA damage in an acute lung injury rabbit model

**DOI:** 10.62675/2965-2774.20250250

**Published:** 2024-12-18

**Authors:** José Roberto Fioretto, Susiane Oliveira Klefens, Mário Ferreira Carpi, Marcos Aurélio Moraes, Rossano César Bonatto, Ana Lúcia Anjos Ferreira, Camila Renata Corrêa, Cilmery Suemi Kurokawa, Carlos Fernando Ronchi

**Affiliations:** 1 Department of Pediatrics Faculdade de Medicina Campus de Botucatu Universidade Estadual Paulista “Júlio de Mesquita Filho” Botucatu SP Brazil Department of Pediatrics, Faculdade de Medicina Campus de Botucatu, Universidade Estadual Paulista “Júlio de Mesquita Filho” - Botucatu (SP), Brazil.; 2 Department of Physical Therapy Universidade Federal de Uberlândia Uberlândia MG Brazil Department of Physical Therapy, Universidade Federal de Uberlândia - Uberlândia (MG), Brazil.

**Keywords:** Respiratory distress syndrome, Lung injury, Respiration, artificial, Carotenoids, DNA damage, Inflammation

## Abstract

**Objective:**

To investigate the effects of lycopene supplementation on inflammation, lung histopathology and systemic DNA damage in an experimentally induced lung injury model, ventilated by conventional mechanical ventilation and high-frequency oscillatory ventilation, compared with a control group.

**Methods:**

Fifty-five rabbits sampled by convenience were supplemented with 10mg/kg lycopene for 21 days prior to the experiment. Lung injury was induced by tracheal infusion of warm saline. The rabbits were randomly assigned to the control group and subjected to protective conventional mechanical ventilation (n = 5) without supplementation or the experimental group that was subjected to acute lung injury and provided conventional mechanical ventilation and high-frequency oscillatory ventilation with and without lycopene supplementation (n = 10 rabbits in each group). Lung oxidative stress and the inflammatory response were assessed based on the number of polymorphonuclear leukocytes in bronchoalveolar lavage fluid, DNA damage and pulmonary histological damage.

**Results:**

A significant worsening of oxygenation and a decrease in static lung compliance was noted in all groups after pulmonary injury induction (partial pressure of oxygen before 451.86 ± 68.54 and after 71 ± 19.27, p < 0.05). After 4 hours, the high-frequency oscillatory ventilation groups with and without lycopene supplementation as well as the group receiving protective conventional mechanical ventilation with lycopene supplementation showed significant oxygenation improvement compared with the protective conventional mechanical ventilation group without supplementation (partial pressure of oxygen of the group with mechanical ventilation without lycopene of 102 ± 42, of the group that received conventional protective mechanical ventilation with lycopene supplementation of 362 ± 38, of the high-frequency group without lycopene supplementation of 420 ± 28 and of the high-frequency group with lycopene supplementation of 422 ± 25; p < 0.05). Compared with rabbits not receiving supplementation, those in the groups that received protective conventional mechanical ventilation with lycopene supplementation and high-frequency oscillatory ventilation with lycopene supplementation had significantly less inflammation as well as less histological injury (p < 0.05). Compared with rabbits subjected to protective conventional mechanical ventilation, significantly lower DNA damage was observed in rabbits supplemented with lycopene (p < 0.05).

**Conclusion:**

Lycopene supplementation reduces inflammatory and histopathological lung injuries, regardless of the associated ventilatory mode. In addition, lycopene improved oxygenation and reduced DNA damage when protective conventional mechanical ventilation was used.

## INTRODUCTION

Acute respiratory distress syndrome (ARDS) is characterized by an inflammatory process leading to alveolar–capillary barrier breakdown, with the development of interstitial and alveolar edema, decreased lung compliance, pulmonary hypertension, ventilation/perfusion ratio imbalance and hypoxemia refractory to oxygen administration. Despite a better understanding of ARDS pathophysiology and consequent advances in therapeutic strategies for ARDS patients, mortality has remained high.^(1)^

Mechanical ventilation (MV) is one of the main ARDS treatments^(2)^ because it is able to modify disease evolution and reduce mortality.^(1,2)^ Although MV is an important ARDS treatment,^(3)^ it can cause lung injury due to the high inspired tidal volume or even the high pressures typically used in conventional MV (CMV).^(4,5)^ Thus, an interesting alternative is high-frequency oscillatory ventilation (HFOV), which uses low volumes and high frequencies, thus avoiding hyperdistention.^6^

Although ARDS pathogenesis is multifactorial, a common denominator and determinant is the presence of oxidative stress. In fact, there is evidence that reactive oxygen species determine vascular endothelial and pulmonary epithelial injury,^(10)^ which is responsible for ARDS development and progression.^(11)^ Thus, it is essential to better understand the role of oxidative stress in the onset and perpetuation of the inflammatory process that occurs in ARDS.^(12)^

Recently, the protective role of natural biochemical antioxidants in foods for preventing oxidative damage caused by free radicals (reactive oxygen species and reactive nitrogen species) has attracted increased interest.^(13)^ Lycopene is a lipophilic carotenoid responsible for the red color of many fruits and vegetables^(14)^ and is abundant in tomatoes and their products.^(15)^ Lycopene exhibits a nonprovitamin A activity that is relatively resistant to heat in tomato processing. Owing to its large number of conjugated double bonds, lycopene is considered one of the best antioxidants among carotenoids. In addition, it is one of the most potent antioxidants found in the human body, with an antioxidant power 100 times greater than that of vitamin E and vitamin C.^(16)^ Thus, the primary aim of the present study was to investigate the effects of lycopene supplementation on inflammation, lung histopathology and systemic DNA damage in an experimentally induced lung injury model, ventilated by CMV and high-frequency oscillatory ventilation, compared with a control group (CG).

## METHODS

The study was approved by the Committee on Ethics in the Use of Animals (CEUA; protocol 1107/2014) and conducted in the laboratory of the Experimental Research Unit (UNIPEX) - *Faculdade de Medicina - Campus de Botucatu* of the *Universidade Estadual Paulista “Júlio de Mesquita Filho”* (UNESP).

### Design, animals, and instrumentation

This was a prospective, controlled, in vivo study using laboratory animals. Norfolk male white rabbits (weighing 2.0 - 3.0kg) were obtained from the UNESP Medical School Animal Facility.

### Lycopene supplementation

The rabbits were adapted to the Experimental Research Laboratory for two weeks. The animals were maintained (one animal/cage) under controlled temperature (22 ± 2ºC) and humidity (70 ± 10%) conditions with a light/dark cycle (12 hours). Filtered water and food (Purina®, Brazil) were provided ad libitum.

After a two-week adaptation period, animals randomly allocated to the supplementation groups received a daily lycopene solution made from the compound Lyc-O-Mato® 6%. Lycopene was diluted in commercial corn oil (Mazola® Brazil) and supplied at 10mg/kg by gavage for three weeks. The animals in the CG received corn oil at the same concentration. The supplemented product contains tomato extract featuring 6% lycopene, as well as other natural tomato phytonutrients (such as tocopherols, phytoene, phytofluene, beta-carotene, phospholipids, and phytosterols) extracted from lycopene-rich tomatoes and produced in accordance with US patent number 5,837,311 and EU patent number 844,831.

Diet intake and animal weight were recorded daily throughout the study period. The temperature, humidity, and light/dark cycle conditions were the same as those used during the adaptation period.

### Group distributions and rabbit treatments

Fifty-five male Norfolk white rabbits were used. Previously, ten healthy animals were subjected to euthanasia to form the baseline groups, and five of these animals received supplementation. The baseline groups included the following groups: baseline without supplementation (BWS, n = 5) and baseline supplemented with lycopene (BS, n = 5). Baseline groups were designed for carotenoid analysis. Five animals in the Control Group (CG) were subjected to protective Conventional Mechanical Ventilation (CMV) without supplementation, comprising the CG (n = 5). The remaining animals were distributed into groups subjected to lung injury and mechanical ventilation treatment as follows: lung injury submitted to protective CMV with lycopene supplementation (CMVL) (n = 10) and without supplementation (CMV) (n = 10) and lung injury submitted to High-frequency Oscillatory Ventilation (HFOV) with lycopene supplementation (HFL) (n = 10) and without supplementation (HF) (n=10).

The rabbits were treated as previously described.^(17,18)^ Briefly, after weighing, the rabbits were anesthetized with ketamine (50mg/kg IM) and xylazine (2mg/kg IM). A tracheotomy was performed by inserting a tracheal tube (3.0 - to 3.5mm ID; Portex, Hythe, UK), which was secured in position with umbilical tape. Immediately after tracheotomy, ventilation was initiated with a Galileo Gold ventilator (Hamilton Medical) with the following parameters: respiratory rate from 25 to 50rpm adjusted according to partial pressure of carbon dioxide (PaCO_2_); tidal volume (VT) 7mL/kg; positive end-expiratory pressure (PEEP) 5cmH_2_O; and plateau pressure limited to (less than or equal to) 30cmH_2_O. These parameters were maintained for a 10-minute stabilization period until lung injury induction.

Anesthesia was maintained with continuous intravenous infusion of ketamine (10mg·kg/hour). Muscle paralysis was induced by intravenous administration of pancuronium bromide (0.2mg/kg), which was maintained at 0.1mg/kg doses as needed to control movement. Euthanasia was performed via the intravenous administration of high doses of ketamine and xylazine. The rabbits were cared for, minimizing discomfort, distress, and pain in accordance with the guidelines of the National Institutes of Health (NIH).

One animal from the CG (refractory hypotension), one from the CMV without lycopene (pneumothorax and tracheal bleeding) group and one from the HFOV with lycopene (refractory hypotension) group died. These animals were not included or analyzed.

### Lung injury induction

Lung injury was induced by lung lavage with 30mL/kg aliquots of 0.9% warm saline solution (38°C) as previously described.^(17,18)^This is one of the most commonly used animal acute lung injury (ALI) models.^(19)^ This model is primarily a surfactant depletion model, which causes lung injury very similar to human ARDS based on its effects on oxygenation, respiratory system compliance, atelectasis, and perivascular/peribronchial edema.

According to the behavior of the variables analyzed in other studies that used similar methodologies, 10 rabbits were studied per treatment group.^(4,17,18)^

### Experimental groups

Rabbits were randomly assigned to one of seven experimental groups: healthy animals without ventilation and without supplementation (BWS: n = 5); healthy animals without ventilation and supplemented with lycopene (BS: n = 5); healthy animals subjected to protective CMV without supplementation (CG: n = 5); animals subjected to lung injury with protective CMV and supplemented with lycopene (CMVL: n = 10); animals with lung injury with protective CMV without supplementation (CMV: n = 10); animals with lung injury with HFOV and supplemented with lycopene (HFL: n = 10); and animals with lung injury and receiving HFOV without supplementation (HF: n = 10).

For the animals in the CMV groups, including those with or without lycopene supplementation, the PEEP was initially maintained at 5cmH_2_O. After lung injury, it was increased to 8cmH_2_O during the first hour and then gradually increased to 10cmH_2_O in the following three hours to minimize systemic hypotension. In the HFOV groups, a mean airway pressure (Paw) of 12 - 14cmH_2_O, respiratory rate of 10Hz, inspiratory time of 33%, and initial pressure amplitude of 20cmH_2_O were maintained using a SensorMedics 3100A ventilator (Viasys Healthcare, Yorba Linda, CA, United States). In the CMV, pressure-regulated volume control mode was used, and the respiratory rate was maintained at 40 - 50 breaths/minute to reach the targeted PaCO_2_ (40 - 45mmHg), as the pressure amplitude in the HFOV was modified to the same PaCO_2_ level. The fraction of inspired oxygen (FiO_2_) was maintained at 1.0 throughout the experiment for all groups. The ventilator settings were chosen according to previous studies by our group.^(17,18,20)^

After lung injury, blood gas measurements were performed every 30 minutes during the 4-hour experimental protocol.

### Study design

**Figure 1:**
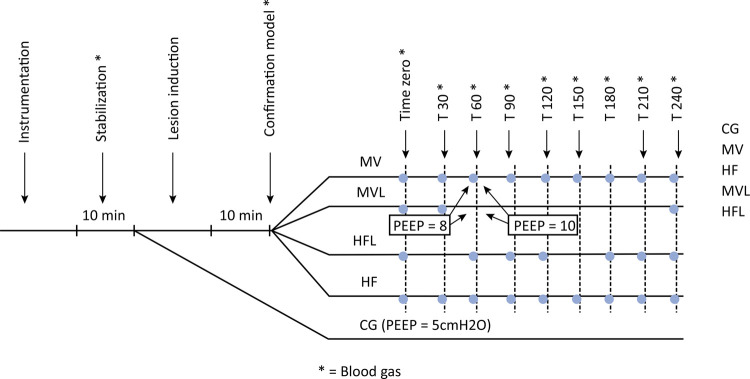
Experimental protocol.

Figure 1 shows the experimental protocol.

T - time; CMV - mechanical ventilation group without lycopene; CMVL - mechanical ventilation with lycopene; HFL - high frequency with lycopene; HF - high frequency without lycopene; CG - control group; PEEP - positive end-expiratory pressure.

### Tissue collection

The endotracheal tube was clamped at the PEEP level during use, and the thorax was carefully opened to observe for signs of pneumothorax to confirm proper catheter placement and to harvest tissue. The animals were exsanguinated before lung removal. The heart and lungs were removed *en bloc* from the thoracic cavity. In each group, the left mainstream bronchus of five rabbits was cannulated, and the left lung was lavaged with two 15mL/kg aliquots of saline. Additionally, left lungs not instrumented for bronchoalveolar lavage collection were removed and used for histopathological analysis.

### Histopathological analysis

The lungs were filled with 10% buffered formalin, and the alveolar architecture was preserved using a slow gravity formalin drip at a maximum pressure of 30cmH_2_O. After at least 48 hours of fixation, the fragments were embedded in paraffin. Then, axial lung sections were generated, stained with hematoxylin and eosin, and blindly examined by two independent pathologists. Ten microscopic fields were randomly selected for examination of each slide, yielding a total of 20 tests for each animal. Pulmonary histological damage was quantified based on a score using seven variables (alveolar and interstitial inflammation, alveolar and interstitial hemorrhage, edema, atelectasis and necrosis). Injury severity was graded for each of the seven variables as follows: no injury = 0, injury to 25% of the field = 1, injury to 50% of the field = 2, injury to 75% of the field = 3, and diffuse injury = 4. The maximum possible score was 28, and the lowest possible score was zero.^(21)^

### Bronchoalveolar lavage

Bronchoalveolar lavage fluid was collected, and the total number of cells was counted using a hemocytometer chamber. The cells were differentiated via a Panotic Staining Kit (Laborclin, Pinhais, Brazil), and the percentage of polymorphonuclear leukocytes was assessed.^(21)^

### Blood collection

The collected blood was transferred to tubes containing ethylenediaminetetraacetic acid (EDTA). These tubes were placed in a centrifuge. After centrifugation (2,500 × g at 4°C for 10 minutes), the obtained plasma was stored in a freezer at -80°C to subsequently measure the concentration of carotenoids. Blood intended for the determination of oxidative DNA damage (comet test) was collected in an EDTA tube and immediately processed.

### Lycopene plasmatic concentration

To confirm lycopene absorption, plasma carotenoid measurements were made using high-performance liquid chromatography (HPLC) according to Correa et al.^(22)^ Briefly, plasma samples (200µL) were extracted with 2mL of chloroform:methanol (2:1) followed by 3mL of hexane. The samples were dried under nitrogen air and resuspended in 75µL of ethanol:methyl tert-butyl ether (2:1), of which 25µL was assessed using HPLC. The HPLC system consisted of a Waters 2695 Separation Module, 2996 Photodiode Array Detector, a Waters 2475 Multi λ Fluorescence Detector, a C30 carotenoid column (3µm, 150 × 3.0mm, YMC, Wilmington, NC), and a Waters Millennium 32 data station. The mobile phase was methanol:methyl tert-butyl ether:water (85:12:3 by volume with 1.5% ammonium acetate in water; solvent A) and methanol:methyl tert-butyl ether:water (8:90:2 by volume with 1% ammonium acetate in water; solvent B). The gradient procedure has been previously reported. The results were adjusted using an internal standard of echinenone. The coefficient of variation (CV) for the interassay (n = 25) was 4%, and that for the intra-assay was 4% (n = 9). The recovery of the internal standard averaged 97%.

### Single-cell gel electrophoresis analysis (comet assay)

DNA strand breaks were measured in cells obtained from lung tissue and peripheral blood via a comet assay as proposed by Singh et al.^(23)^and Tice et al.,^(24)^ with some modifications. Volumes of 20µl of cells obtained from lung tissue homogenate or peripheral blood were added to 150µl of 0.5% low-melting-point agarose at 37°C. From the mixtures, 130µl was layered onto slides precoated with 1.5% normal agarose, covered with a coverslip, and left for 5 minutes at 4°C to solidify the agarose. Subsequently, the coverslips were carefully removed, and the slides immersed in a lysis solution for 24 hours. Then, slides were washed with phosphate buffered saline (PBS) for 5 minutes and immersed in a freshly prepared alkaline buffer [1mM EDTA, 300mM NaOH (pH > 13)] in a horizontal electrophoresis tray. After a 40-min DNA unwinding period, electrophoresis was conducted at 25 V and 300 mA for 30 min, followed by 15 minutes neutralization with 0.4 M Tris buffer (pH 7.5). The slides were washed in absolute ethanol and stored at room temperature until drying. The gel on each slide was stained with 50µl of SYBR Green (Trevigen, Gaithersburg, MD) and diluted 1:10,000 in Tris-EDTA buffer. The slides were examined via a fluorescence microscope at 400x magnification.^(25)^ Every step was performed under indirect light. The slides were coded and analyzed without knowledge of the sample identity. DNA damage was measured as the percentage of DNA in the tail using a computer scoring system (comet assay IV Software, Perceptive Instruments, Haverhill, Suffolk, UK).

### Statistical analysis

The data were analyzed with SigmaPlot 11 (Chicago, Illinois, United States). Normally distributed data were compared between the different treatment groups using factorial analysis of variance with all pairwise comparison procedures (Student–Newman–Keuls test) and are expressed as the mean ± standard deviation (SD). Data showing a nonnormal distribution were compared by Kruskal–Wallis factorial analysis of variance on ranks with all pairwise comparisons by Dunn’s test and expressed as medians (ranges). Intragroup comparisons at different time points were performed via Friedman’s repeated measures analysis of variance on ranks with all pairwise multiple comparison procedures via Dunnett’s method and Tukey´s multiple test. Contingency tables of categorical data were compared via Fisher’s exact test. Repeated-measures ANOVA complemented with the Bonferroni multiple comparisons test was used to compare independent groups. Statistical significance was defined as p ≤ 0.05.

## RESULTS

The serum lycopene concentrations are shown in table 1. The results revealed that lycopene supplementation significantly increased the plasma lycopene concentration.

**Table 1:** Lycopene plasma concentration (*µg*/dL)

Group	Without supplementation	With supplementation
CMV	0	1,272
HF	0	1,024
Baseline (BWS/BS)	0	1,039

CMVG - conventional mechanical ventilation; HF - high-frequency oscillatory ventilation group; Baseline: BWS (without lycopene supplementation) and BS (without lycopene supplementation). p < 0,05. The data are expressed as the means ± standard deviations.

There was no significant difference between the groups in terms of weight (2.6 ± 0.2kg; p > 0.05) or the number of lung lavages used to induce ALI (average of 6/group, p > 0,05).

After lung injury induction, significant hypoxemia and poorer ventilation were observed, with worsening respiratory system compliance and oxygenation (Table 2). There was no significant difference in the mean arterial pressure among the groups. Vasoactive drug support pressure was used if necessary to maintain the mean arterial pressure.

**Table 2:** PaO2/FiO2 and pulmonary compliance comparisons before and after acute lung injury induction

	CG n = 5	CMV n = 10		HF n = 10		CMVL n = 10		HFL n = 10	
	Baseline	Before	After	Before	After	Before	After	Before	After
PaO_2_/FiO_2_	469.2 ± 56.1	377.4 ± 49.7	68.0 ± 18.1*	474.5 ± 86.7	67.4 ± 18.4*	461.9 ± 57.9	70.0 ±2 1.3*	476.3 ± 92.3	78.6 ±19.3*
Pulmonary compliance (mL/cmH_2_O)	2.50 ± 0.77	2.09 ± 0.41	0.68 ± 0.36*	2.50 ± 0.38	0.46 ± 0.25*	3.20±0.63	0.63 ± 0.38*	2.36 ± 0.41	0.66 ± 0.35*

PaO_2_/FiO_2_ - ratio; CMV - conventional mechanical ventilation without supplementation; HF - high-frequency oscillatory ventilation without supplementation; CMVL - conventional mechanical ventilation supplemented with lycopene; HFL - high-frequency oscillatory ventilation supplemented with lycopene. The ANOVA repeated measures model in independent groups was complemented with the Bonferroni multiple comparisons test. * p < 0.05. The data are expressed as the means ± standard deviations.

**Figure 2:**
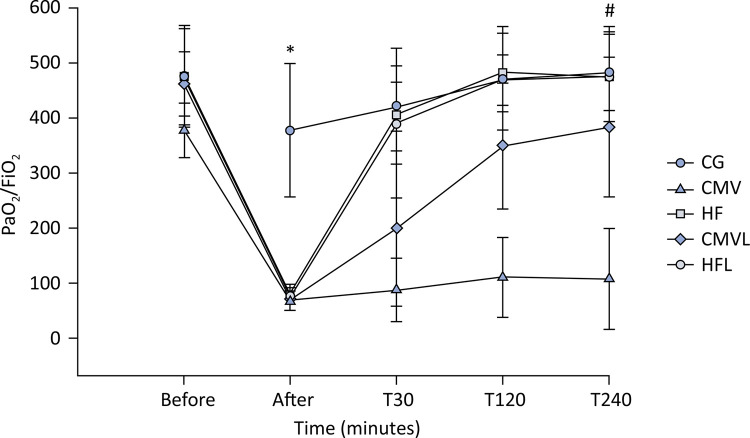
Time course of the PaO2/FiO2 ratio during the 4-hour experimental period.

Compared with those at baseline, the groups subjected to lung injury presented significant hypoxemia. After 4 hours of MV, the HFOV, lycopene-free, and protective CMV with lycopene groups presented significant improvements in oxygenation compared with the protective CMV group without supplementation, with a PaO_2_/FiO_2_ ratio similar to that noted at baseline and comparable to that of the CG, as shown in figure 2. In addition, the CG and HFOV groups exhibited improved in oxygenation after 30 minutes of MV (T30).

Statistical tests: Repeated-measures ANOVA in independent groups complemented with the Bonferroni multiple comparisons test. * p < 0.05 at T0, comparing the control group with the other groups; # p < 0.05 at T240, comparing the control group, high-frequency with lycopene, high-frequency without lycopene, and mechanical ventilation with lycopene groups with the mechanical ventilation group without lycopene. CG - control group; CMV - mechanical ventilation group without lycopene; HF - high-frequency group without lycopene; CMVL - mechanical ventilation with lycopene; HFL - high-frequency with lycopene.

**Figure 3:**
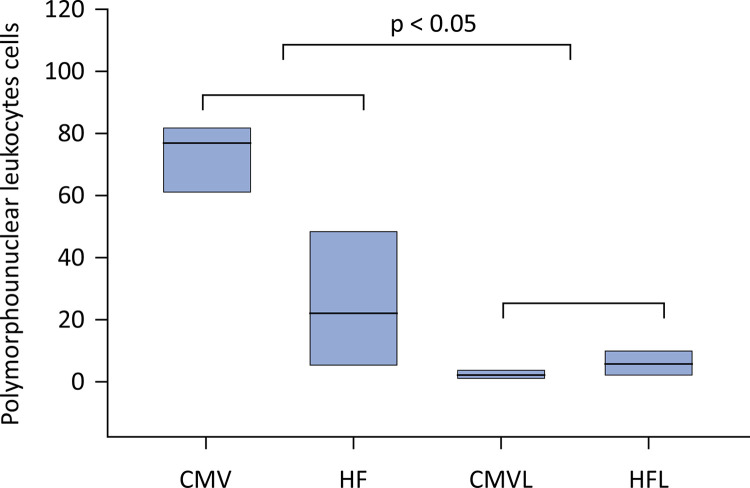
Polymorphonuclear leukocyte cells recovered from bronchoalveolar lavage fluid.

Recovery of polymorphonuclear leukocytes from bronchoalveolar lavage fluid showed that the groups supplemented with lycopene presented significantly lower values than did the animals without supplementation, as shown in figure 3.

There was no difference between mechanical ventilation group without lycopene (76.5 ± 17.5) and high-frequency oscillatory ventilation (22 ± 38) or between conventional mechanical ventilation group (2 ± 1.5) and high-frequency (5.5 ± 7.5), p > 0.05. However, the groups supplemented with lycopene presented a lower number of polymorphonuclear leukocytes, ‘a’ *versus* ‘b’, p < 0,05. CMV - conventional mechanical ventilation group without supplementation; HF - high-frequency oscillatory ventilation group without supplementation; CMVL - conventional mechanical ventilation group supplemented with lycopene; HFL - high-frequency oscillatory ventilation group supplemented with lycopene. Statistical test: Kruskal–Wallis analysis of variance complemented by Dunn’s multiple comparisons test.

Compared with the control group and the lycopene-supplemented groups, the lycopene-supplemented groups presented reduced histopathological injury scores (Figures 4A and 4B).

**Figure 4A:**
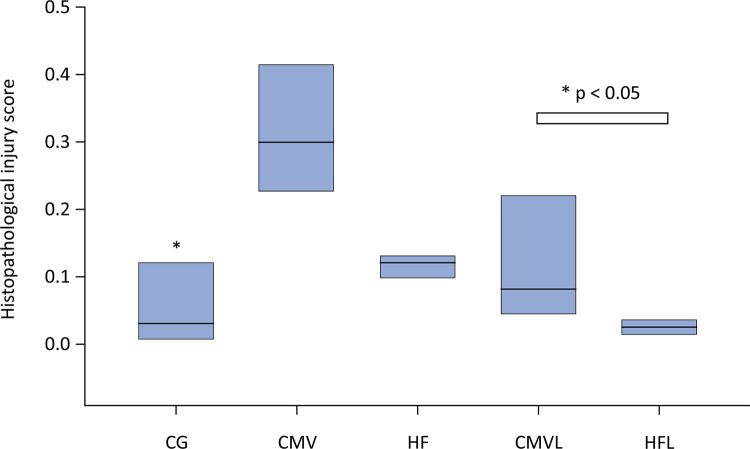
Histopathological injury score in lung tissue.

CG - control group; CMV - conventional mechanical ventilation group without supplementation; HF - high-frequency oscillatory ventilation group without supplementation; CMVL - conventional mechanical ventilation group supplemented with lycopene; HFL - high-frequency oscillatory ventilation group supplemented with lycopene. Statistical test: Kruskal–Wallis nonparametric ANOVA complemented with Dunn’s multiple comparisons test. The data are expressed in boxplots, and their median values are shown. Compared with conventional mechanical ventilation group and high-frequency without supplementation, control group, high-frequency oscillatory ventilation group supplemented with lycopene and conventional mechanical ventilation group supplemented with lycopene presented significantly lower values (p < 0.05).

**Figure 4B:**
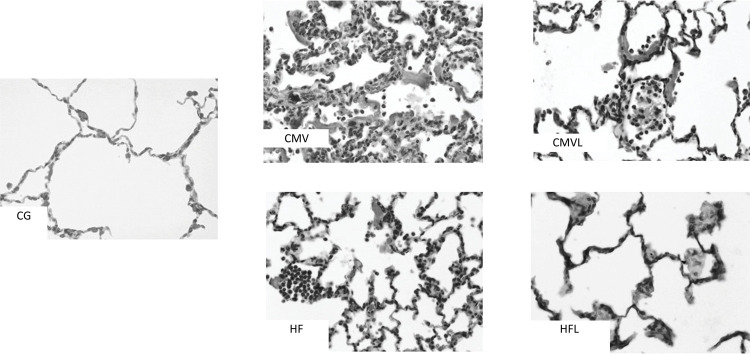
Optical microscopy digital photomicrographs (200x, hematoxylin and eosin) of representative samples of lung-dependent areas.

CG - control group; CMV - conventional mechanical ventilation group without supplementation; CMVL - conventional mechanical ventilation group supplemented with lycopene; HF - high-frequency oscillatory ventilation group without supplementation; HFL - high-frequency oscillatory ventilation group supplemented with lycopene. Data are shown as the median (range). Kruskal–Wallis analysis of variance was used, with subsequent comparisons by the Dunn test.

**Figure 5:**
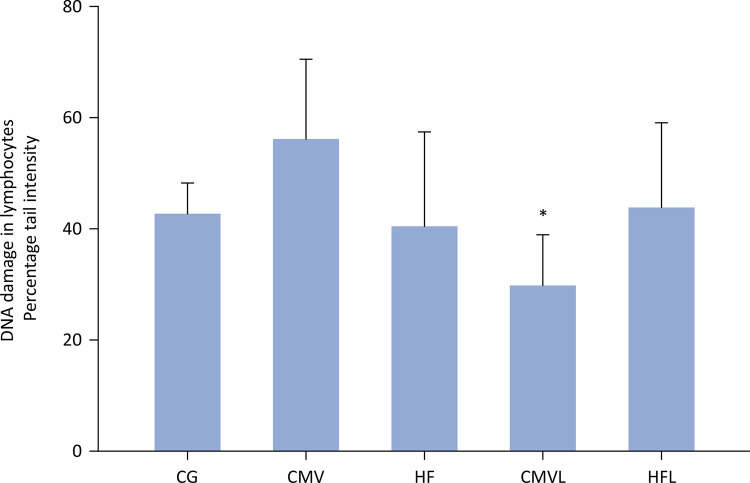
DNA damage in lymphocytes, evaluated by the percentage of DNA in the tail (comet test - tail intensity).

DNA damage analysis of lymphocytes revealed no significant difference between the different MV groups. However, a significant difference was noted when comparing animals with CMV with and without lycopene supplementation. Among the CMV groups, DNA damage was significantly reduced in animals supplemented with lycopene (CMVL 29.71 ± 9.21 < CMV 56.07 ± 14.39; p < 0.05) than in those without lycopene supplementation, as shown in figure 5.

CG - control group; CMV - conventional mechanical ventilation group without supplementation; HF - high-frequency oscillatory ventilation group without supplementation; CMVL - conventional mechanical ventilation group supplemented with lycopene; HFL - high-frequency oscillatory ventilation group supplemented with lycopene. Statistical test: analysis of variance complemented with Tukey’s multiple comparisons test. The data are expressed as the means ± standard deviations. *p < 0.05 between conventional mechanical ventilation group without supplementation and conventional mechanical ventilation group supplemented with lycopene.

## DISCUSSION

The numerous benefits of lycopene,^(26,27)^ such as its anti-inflammatory activity and antioxidant capacity, have been reported previously. Recently, some studies have shown that lycopene has a potent antioxidant capacity and protective effect on cells through the suppression of oxidative damage. Lycopene also reduces the levels of inflammatory biomarkers in cells through its antioxidant activity.^(28)^ Although it is considered one of the best carotenoids because of its ability to act as a free radical scavenger,^(29)^ few studies have evaluated the role of lycopene in experimental ALI.

Most studies have focused on the imbalance of proinflammatory/anti-inflammatory mediators and oxidation/reduction. Carotenoids are known to suppress oxidative damage by activating antioxidant enzymes and stimulating the immune system. It has been reported that ALI involves superoxide radicals and inflammatory processes; since lycopene is a member of the carotene family, it can prevent the mechanism of oxidative damage in lung injury and efficiently quench singlet oxygen species.^(14,15)^

In a previous study from our group, we demonstrated that oxidative stress was present in this experimental ALI model and that there was greater antioxidant defense and lower DNA damage in response to HFOV.^(18)^ On the basis of the protective effects of HFOV on ARDS oxidative metabolism, as well as the powerful role of lycopene as an antioxidant, we hypothesized that lycopene could exert a protective effect in an ALI experimental model induced in rabbits in the present study.

Previous clinical and experimental studies have demonstrated that, compared with CMV, HFOV improves oxygenation.^(30-32)^In the present study, we observed that animals previously supplemented with lycopene and subjected to CMV presented oxygenation levels similar to that of animals subjected to HFOV, demonstrating that lycopene administration improved oxygenation, regardless of which MV mode was used. In addition, compared with the HFOV group, the CMVL group showed rapid recovery, improving hypoxemia after lung injury induction. Owing to the lack of studies in this area, it is difficult to compare our results.

Interestingly, lycopene supplementation did not have an additional advantage in terms of oxygenation at the end of the experiment among the groups under HFOV, indicating that this MV mode achieves consistent benefits by itself.

In the ANTIOZ study, the authors evaluated the ability of lycopene supplementation to protect the lungs from ozone exposure. A randomized clinical trial revealed improvements in lung function in individuals who ingested vegetable juice compared with those in the placebo group, thus providing protection against the adverse effects of ozone.^(33)^ These results could be explained by the action of lycopene in the lung epithelial lining fluid, as well as inside the cells, poviding additional protection.^(34)^

A recent review by Cesta et al.,^(35)^ investigating the role of IL-8 in lung inflammation and disease pathogenesis suggested that IL-8 is a possible new therapeutic target that can efficiently modulate the hyperinflammatory response in ARDS. Lycopene has potential effects against inflammation,^(36,37)^and investigating how lycopene could play an important role in inhibiting IL-8 expression would be interesting.

In our study, we evaluated inflammation based on polymorphonuclear cell counting (PMN) in BAL fluid and found that rabbits supplemented with lycopene had significantly less inflammation than did rabbits in the HFOV and CMV groups without supplementation. In a recent study by our group,^(20)^we compared neutrophil counts among three ALI experimental groups: a CG without lung injury subjected to CMV; an ALI group subjected to HFOV; and an ALI group subjected to CMV. The ALI groups were maintained in the prone position. No significant differences were noted between the ALI groups; however, these animals had higher values compared to control group. On the basis of the findings of the present study and previous studies, we suggest that lycopene is an important tool for reducing lung inflammation.

To confirm the anti-inflammatory role of lycopene, Bae et al.^(38)^ evaluated the protective role of carotenoids in primary endothelial human umbilical vein cells activated by lipopolysaccharides (LPS). This study demonstrated the inhibition of LPS-induced proinflammatory responses with lycopene and increased alveolar capillary barrier integrity from a stage prior to aggression.

Over the last few years, studies have shown that oxidative stress is associated with severe diseases in critically ill patients.^(39)^ This occurs especially because tissue perfusion decreases, with an increase in the oxidant amount present in the body.^(40)^Some epidemiological studies highlight the importance of the intake of carotenoids, especially lycopene and b-carotene, demonstrating an association between higher carotenoid concentrations and a lower risk of disease. This is due mainly to the antioxidant properties of lycopene, but other mechanisms, such as cell growth regulation, gene expression modulation and the immune response, are also potentially involved.^(41)^

In our study, the groups with lycopene supplementation presented less histological lung injury compared to the groups without supplementation. To the best of our knowledge, no reports have evaluated lung histological damage related to lycopene supplementation in experimental ALI. Interestingly, our results showed that lycopene supplementation and the use of protective CMV led to biochemical and histopathological benefits similar to those exhibited by HFOV alone in this lung injury experimental model.^(21,22)^

In this study, DNA damage was assessed based on DNA strand breaks. This analysis is not specific for detecting oxidative stress; however, we know that DNA damage can also occur due to the overproduction of reactive oxygen species that result in oxidative stress.^(42)^ The CMV supplemented with lycopene significantly reduced damage compared with that in the group without supplementation, and this effect was achieved using the same ventilatory mode. This result suggests that lycopene supplementation may be an additional adjuvant therapy to protect against CMV, as it is considered a very effective antioxidant.^(43)^

Although many studies have evaluated severe ARDS outcomes as well as advances in treatments, especially mechanical ventilation support^(23,24)^and prone positioning,^(25-27)^ few studies have evaluated the relationship between lycopene and lung oxidative protection. Similarly, few studies have evaluated antioxidant dosages in critically ill patients to determine the ideal dose for different nutritional therapies, such as enteral and parenteral nutrition.

Tomato, a natural carotenoid source, is one of the most consumed fruits worldwide^(29)^ and has well-defined biochemical characteristics,^(34)^ with an important biological role in the prevention and treatment of many severe syndromes. However, lycopene absorption depends on factors such as temperature and cooking time, as well as the presence of lipids or liposoluble compounds.^(42)^

### Study limitations

This ALI model has several limitations. One limitation is the experimental duration, which is limited to four hours, in view of the rabbits’ viability. In addition, animals were subjected to FiO_2_ - 1.0 during this period, a factor that potentially causes lesions in the lung parenchyma, which interfere with oxidative metabolism. However, the same oxygen concentration was used for all groups at the same time, possibly excluding variations between groups due to similar levels of oxygen toxicity. Another limitation is that we were not able to measure the concentrations of cytokines, such as IL-8, and oxidative stress markers.

## CONCLUSION

Lycopene supplementation reduces inflammatory and histopathological lung injuries, regardless of the associated ventilatory mode. In addition, lycopene improved oxygenation and reduced DNA damage when protective conventional mechanical ventilation was used. Given that lycopene is a potent carotenoid found in tomato products, more experimental and clinical studies should be performed in the future to assess its effects in clinical practice.
